# Klinische Untersuchung und Bildgebung bei patellofemoraler Arthrose

**DOI:** 10.1007/s00132-025-04655-2

**Published:** 2025-04-30

**Authors:** Paul Nardelli, Armin Runer

**Affiliations:** 1 Universitätsklinik für Orthopädie und Traumatologie, Medizinische Universität Innsbruck, Anichstraße 35, 6020 Innsbruck, Österreich; 2 Sektion Sportorthopädie Klinikum rechts der Isar, Technische Universität München, München, Deutschland

**Keywords:** Vorderer-Knieschmerz-Syndrom, Diagnostische Bildgebung, Palpation, Patellofemoralgelenk, Körperliche Untersuchung, Anterior knee pain syndrom, Diagnostic imaging, Palpation, Patellofemoral joint, Physical examination

## Abstract

Die patellofemorale Arthrose äußert sich durch vordere Knieschmerzen, verstärkt bei Belastungen wie Treppensteigen, Hocken oder langem Sitzen. Klinisch relevante Zeichen sind belastungsabhängige Schmerzen, Steifheit, Bewegungseinschränkungen, Schwellungen und Krepitation. Die Untersuchung umfasst die Inspektion von Gangbild, Beinachsen, Muskelatrophien und Fehlstellungen sowie Palpation und spezielle Tests wie den „Hyperpression-Test“. Bildgebende Verfahren wie Röntgen, MRT und CT liefern wichtige Informationen zu Knorpelschäden, Maltracking und subchondralen Veränderungen. Ein strukturierter diagnostischer Ansatz ermöglicht eine präzise Einschätzung der Erkrankung und bildet die Grundlage für eine stadiengerechte Therapie.

## Abstract (en)

Patellofemoral osteoarthritis presents with anterior knee pain, exacerbated by activities such as stair climbing, squatting, or prolonged sitting. Clinically relevant signs include load-dependent pain, stiffness, restricted range of motion, swelling, and crepitus. The examination includes gait analysis, inspection of limb alignment, muscle atrophy, and deformities, along with palpation and specific tests such as the “hyperpression test.” Imaging techniques like X‑ray, MRI, and CT offer valuable information on cartilage damage, maltracking, and subchondral changes. A structured diagnostic approach allows precise assessment of the condition and forms the basis for stage-appropriate therapy.

Die patellofemorale Arthrose ist eine häufige Ursache für vorderen Knieschmerz, der durch Alltagsaktivitäten wie Treppensteigen, Hocken oder langes Sitzen verschärft wird und oft mit funktionellen Einschränkungen einhergeht. Dieser Artikel liefert eine evidenzbasierte Übersicht über die diagnostischen Strategien, einschließlich klinischer Untersuchung und bildgebender Verfahren, und bietet praxisrelevante Ansätze für die differenzierte Beurteilung im orthopädischen und sportmedizinischen Kontext.

## Patellofemorale Arthrose

Zu den häufigsten Beschwerden von Personen mit patellofemoraler Arthrose gehört der vordere Knieschmerz, aggraviert durch funktionale Belastung z. B. beim Treppen auf- oder absteigen, Aufstehen aus dem Sitzen, langem Sitzen („movie-theatre-sign“), Knien oder Hocken [1, 2]. Viele Patienten berichten außerdem über Steifheit und Krepitation [2].

Im Rahmen der Anamnese und klinischen Untersuchung soll nach typischen klinischen Merkmalen einer Arthrose gesucht werden, wie fortgeschrittenem Alter, belastungsabhängigen Schmerzen, in schwereren Stadien auch Ruhe- und Nachtschmerzen, Steifheit und Anlaufschmerz, Einschränkung des Bewegungsumfangs und feste (Gelenksveränderungen) und weiche (sekundäre Synovitis) Schwellung, sowie Krepitation [3]. Benachbarte Gelenke sollen in die klinische Beurteilung miteingeschlossen werden und die Beurteilung im Seitenvergleich wird empfohlen.

Anhand der Kriterien von Willy et al. [4] kann die Diagnose eines patellofemoralen Schmerzes gestellt werden, sofern ein retro- oder peripatellärer Schmerz vorliegt, der sich durch Aktivitäten wie Hocken, Treppensteigen, längeres Sitzen oder andere funktionelle Belastungen des patellofemoralen Kompartiments in gebeugter Knieposition reproduzieren lässt. Voraussetzung ist zudem der Ausschluss aller anderen Bedingungen, die vordere Knieschmerzen verursachen könnten.

## Stand- und Gangbild

Die Beurteilung beginnt bereits beim Eintreten des Patienten durch die Beobachtung des Gangbildes und der Körperhaltung. Die Analyse des Gangbildes kann wertvolle Hinweise auf funktionelle Defizite, Schmerzempfinden sowie muskuläre Schwächen liefern. Zudem können abnorme Bewegungsmuster der unteren Extremitäten einen Einfluss auf patellofemorale Beschwerden ausüben [5].

Die Inspektion umfasst eine umfassende Bewertung des Kniegelenks sowohl im Stehen (barfuß) als auch in Rückenlage. Dabei wird neben dem Kniegelenk und der Patella selbst auf potenzielles Übergewicht, Beinlänge, Beinachse, Torsionsdeformitäten, Muskelatrophien sowie Fußfehlstellungen geachtet [6, 7].

## Inspektion und Palpation

**Abb. 1:**
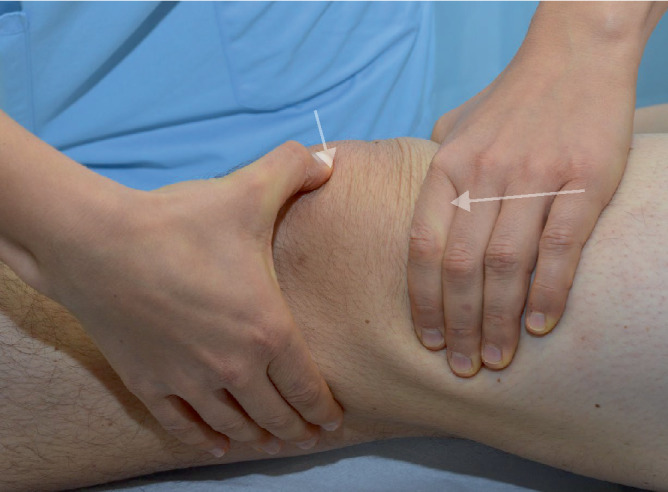
Überprüfung auf eine „tanzende Patella“ durch Druck auf die Patella, nachdem der Recessus suprapatellaris mit der anderen Untersuchungshand von proximal ausgestrichen wurde

Visuell lassen sich Rötungen, Schwellungen, Deformitäten und Narben als mögliche Hinweise auf Verletzungen oder frühere operative Eingriffe identifizieren. Ebenso kann die Position sowie eine etwaige Fehlstellung der Patella beurteilt werden; beispielsweise können ein Tief- oder Hochstand mit bestimmten Pathologien assoziiert sein [8]. Rötungen und Überwärmung deuten auf einen entzündlichen Prozess hin, und bei starker Ausprägung ist insbesondere ein septisches Geschehen differenzialdiagnostisch unbedingt auszuschließen. Krepitationen und Gelenksergüsse können zusammen mit vorderem Knieschmerz auf eine patellofemorale Arthrose hindeuten [2, 7]. Das Ausstreichen des Recessus suprapatellaris durch den Untersucher unterstützt den Nachweis eines Kniegelenksergusses. Hierbei kann das Zeichen einer „tanzenden Patella“ beobachtet werden, bei dem die Patella durch den im femoropatellaren Gelenkspalt konzentrierten Erguss nach vorne angehoben wird (Abb. 1).

Die Palpation der umliegenden Strukturen wie Haut, Retinakula, Quadrizeps- und Patellarsehne mit ihren entsprechenden Ansätzen dient der Differenzierung einer weichteiligen Schmerzgenese [1, 6].

**Abb. 2:**
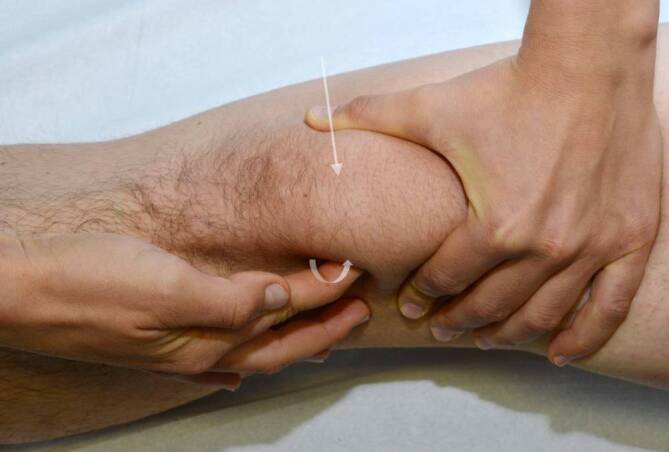
Facettendruckschmerztestung

Um die Druckempfindlichkeit der patellaren Facetten zu beurteilen, wird die Patella nach medial und lateral verschoben (Abb. 2). Dabei ist zu beachten, dass durch das Retinakulum und die Synovia hindurch getastet wird, welche verzerrend eigenständige Schmerzauslöser sein können [6].

## Bewegungsumfang, Patellatracking, Q-Winkel und J‑Sign

Zusätzlich zur detaillierten Untersuchung des patellofemoralen Kompartiments sollte der Bewegungsumfang („range of motion“ [ROM]) des Kniegelenks im Vergleich zur Gegenseite genau evaluiert werden. Das Tracking der Patella lässt sich sowohl dynamisch beim Übergang in die Hocke als auch in Rückenlage des Patienten beobachten und beurteilen. Die Mobilität der Patella sollte idealerweise in Rückenlage bei gestrecktem Bein und entspannter Muskulatur im Vergleich zur Gegenseite untersucht werden, um potenzielle Unterschiede und Auffälligkeiten zu erfassen [6].

Ein Zusammenhang zwischen dem Q‑Winkel und patellofemoralen Beschwerden ist nicht eindeutig belegt

Der Q‑Winkel (Quadrizepswinkel), gemessen von der Spina iliaca anterior superior zur Patellamitte und weiter zur Tuberositas tibiae, wird je nach Quelle entweder im Stehen oder in Rückenlage bestimmt. Dabei können verschiedene Faktoren die Messung beeinflussen, und die Referenzbereiche variieren entsprechend [6]. Ein direkter Zusammenhang zwischen dem Q‑Winkel und patellofemoralen Beschwerden ist wissenschaftlich nicht eindeutig belegt und bleibt umstritten [6]. Ein „J-Sign“, das während der initialen Flexion auftritt, weist auf ein pathologisches Trackingmuster der Patella hin und wird häufig bei Patellainstabilitäten aufgrund hochgradiger Trochleadysplasie beobachtet [6, 9].

## Kraftfähigkeit

**Abb. 3:**
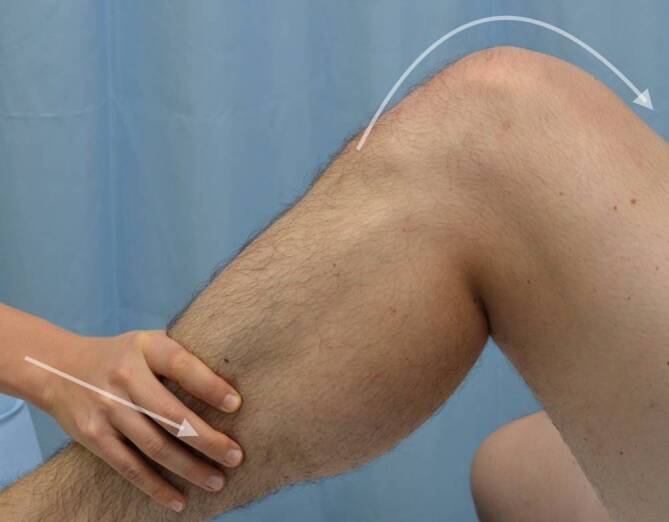
Strecktest gegen Widerstand

Die Streckkraft des Kniegelenks wird im Seitenvergleich sowohl aus der Beugung gegen Widerstand als auch in gestreckter Position getestet, wobei auf etwaig auftretende Beschwerden geachtet wird (Abb. 3). Schmerzen beim Strecken gegen Widerstand weisen häufig auf fokale Knorpelschäden im Bereich der Patella oder der Trochlea oder auf eine beginnende oder fortgeschrittene Patellofemoralarthrose hin. Die gezielte Überprüfung der Quadrizepskraft ist von besonderer Bedeutung, da diese Muskelgruppe eine zentrale Rolle als Stabilisator der Patella einnimmt [7].

## Spezialtests

**Abb. 4:**
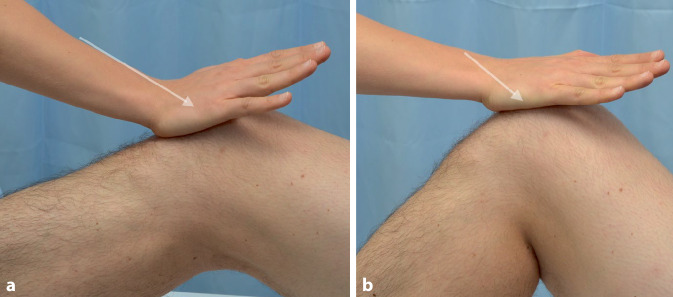
Hyperpression-Test in verschiedenen Flexionsgraden

Beim Hyperpression-Test wird die Patella in verschiedenen Flexionsgraden gegen das Femur gedrückt (Abb. 4). Auftretende Schmerzen können ein Hinweis für Pathologien in den entsprechenden femoropatellaren Gelenksabständen sein [6].

**Abb. 5:**
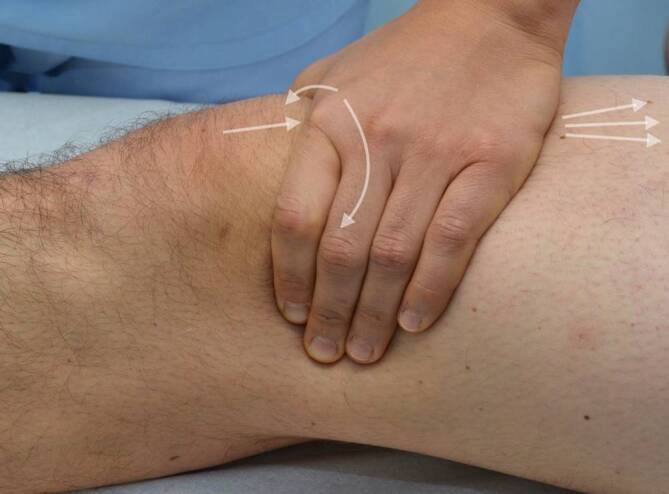
Zohlen-Test

Die dynamische Kompression der Patella gegen das Femur unter gleichzeitigem aktivem Anspannen der Quadrizepsmuskulatur, wie etwa beim Zohlen- oder Clarke-Test, wird laut Post et al. als weniger aussagekräftig betrachtet (Abb. 5). Dies liegt daran, dass hierbei auch peripatelläre Weichteile unter Spannung geraten, welche die Testergebnisse verfälschen können [6].

**Abb. 6:**
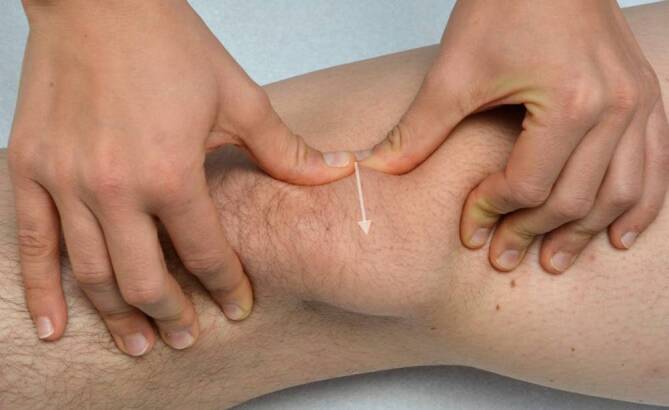
Apprehension-Test

Der Patella-„Apprehension-Test“ gilt als positiv, wenn der Patient bei passiver Lateralisierung der Patella während der klinischen Untersuchung unmittelbar Instabilitätssymptome wahrnimmt und spontan mit einer Abwehrhaltung („apprehensive“) reagiert (Abb. 6). Typischerweise gibt der Patient Beschwerden an und spannt reflexartig die Quadrizepsmuskulatur an, um die Patella dadurch zu rezentralisieren [6, 7].

## Radiologische Abklärung

### Röntgen

Routinemäßige radiologische Abklärung umfasst belastungsabhängige anteroposteriore (a. p.), laterale und axiale Kniegelenksröntgen zur Beurteilung des Ausmaßes der Gelenksdegeneration. Dabei können klassische Arthrosezeichen wie Gelenkspaltverschmälerung, Osteophyten, subchondrale Zysten und Sklerosierung auffällig werden. Die laterale Ansicht erlaubt zudem die Beurteilung der Patellahöhe z. B. mittels Caton-Deschamps-Index sowie einer etwaigen Trochlea- und femoralen Kondylendysplasie.

**Abb. 7:**
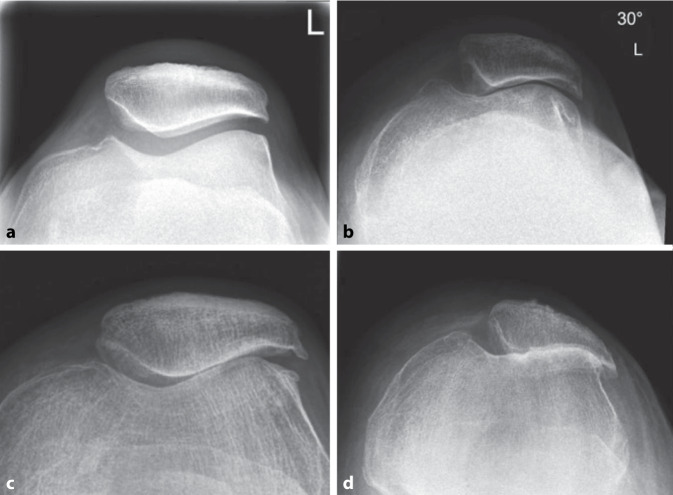
Iwano-Klassifikation von Stadium 1 bis 4 zur radiologischen Beurteilung der patellofemoralen Arthrose: **a** Stadium 1, **b** Stadium 2, **c** Stadium 3, **d** Stadium 4. (Aus [11] Mit freundl. Genehmigung, © 2017 AGA-Komitee-Knie-Patellofemoral)

Zur Beurteilung der patellofemoralen Degeneration spielt die axiale Ansicht (Merchant- oder Sunrise-Ansicht) eine bedeutende Rolle. Zusammen mit der lateralen Ansicht ermöglicht sie eine umfassende Beurteilung der patellofemorale Arthrose . Diese wird nach Iwano [10] in vier Stadien eingeteilt (Abb. 7):

- Stadium 1: geringe Verschmälerung des Gelenkspalts (> 3 mm an der engsten Stelle)
- Stadium 2: moderate Verschmälerung des Gelenkspalts (< 3 mm an der engsten Stelle)
- Stadium 3: Knochen-Knochen-Kontakt auf ≤ 25 % der Gelenkflächen
- Stadium 4: Knochen-Knochen-Kontakt auf > 25 % der Gelenkflächen

Zudem kann die Ausrichtung der Patella in Bezug auf die Trochlea (patellofemorales Alignment) im Sinne eines Patella-Shift beurteilt werden. Dabei wird der Abstand zwischen dem höchsten Punkt der Patella und dem tiefsten Punkt der Trochlearinne parallel zu einer Linie zwischen der medialen und lateralen Trochleaschulter gemessen. Die Kippung der Patella wird durch den Patella-Tilt-Winkel definiert, welcher den Winkel zwischen der Patella und der Trochlealinie beschreibt [12]. Beide Indizes spielen eine wichtige Rolle zur Beurteilung eines möglichen Maltrackings oder zur indirekten Beurteilung vorhandener Weichteilkontrakturen.

### CT und MRT

Mithilfe der Computertomographie (CT) und der Kernspintomographie (MRT) können die drei wichtigsten anatomischen Instabilitätsfaktoren, die laterale Trochleainklination (LTI), die Trochleadysplasie sowie ein pathologischer TTTG- bzw. TTPCL-Abstand bestimmt werden.

Die MRT (Magnetresonanztomographie) ermöglicht in der Diagnostik der patellofemoralen Arthrose und der Beurteilung von Knorpelschäden im patellofemoralen Gelenk eine detaillierte Darstellung der knöchernen und weichteiligen Strukturen des Kniegelenks, einschließlich des patellofemoralen Kompartiments [13]. Sie bietet eine präzise Beurteilung des Knorpelzustands und der subchondralen Knochenveränderungen. Zudem können begleitende Weichteilveränderungen, wie Entzündungen oder Ergüsse, identifiziert werden. Dies ist besonders wichtig, da die klinische Präsentation der patellofemoralen Arthrose variabel sein kann und die MRT dabei hilft, das Ausmaß der degenerativen Veränderungen zu quantifizieren.

Bei Verdacht auf fokale Knorpelschäden im patellofemoralen Gelenk ist die MRT die bevorzugte Bildgebungsmodalität. Sie ermöglicht die Identifizierung von Läsionen, deren Größe, Tiefe und genaue Lokalisation. Dies ist entscheidend für die Planung therapeutischer Maßnahmen, sei es konservativ oder operativ. Zudem können durch die MRT auch begleitende Pathologien, wie subchondrale Ödeme oder Zysten, erkannt werden, die Einfluss auf die Prognose und Therapieentscheidung haben.

MRT-Techniken wie T2-Mapping und T1rho-Imaging können frühe degenerative Knorpelveränderungen im patellofemoralen Gelenk sichtbar machen, noch bevor morphologische Defekte in herkömmlichen Sequenzen erkennbar sind [14].

Die Einteilung von Knorpelschäden kann z. B. angelehnt an die Klassifikation der ICRS (International Cartilage Repair Society) erfolgen oder z. B. über den Amadeus-Score erfolgen:

Der *A*rea *M*easurement *A*nd *DE*pth & *U*nderlying *S*tructures (AMADEUS) Score ist ein Klassifikations- und Bewertungssystem zur Beurteilung der Schwere von fokalen, osteochondralen Defekten im Kniegelenk mittels MRT [15]. Er berücksichtigt die Parameter Knorpeldefektgröße in cm^2^, Defektausdehnung in die Tiefe und Mitbeteiligung des subchondralen Knochens, sowie Knochenmarködem. Diese Parameter sind punktegewichtet und können 0 (schlimmstenfalls) bis 100 (bestenfalls) Punkte ergeben und werden in vier Grade I (> 75 Punkte) bis IV (≤ 25 Punkte) unterteilt [15]. Der AMADEUS-Score ermöglicht durch seine standardisierte Bewertung eine objektive Einschätzung des Schweregrades von Knorpelschäden und dient als wichtige Grundlage für Therapieentscheidungen. Obwohl der AMADEUS-Score eine präzise Einschätzung des Knorpelschadens bietet, zeigt er lediglich eine begrenzte Korrelation mit dem zu erwartenden postoperativen Ergebnis [16–18].

## Fazit für die Praxis

- *Symptome und klinische Zeichen:* Anteriorer Knieschmerz, häufig belastungsinduziert (Treppensteigen, Hocken, langes Sitzen), oft begleitet von Steifheit, Krepitation und Bewegungseinschränkungen.
- *Klinische Untersuchung:* Systematische Evaluation durch Inspektion (Achse, Atrophien, Deformitäten), Palpation (Druckschmerz, Erguss) und spezifische Tests wie den Hyperpression-Test.
- *Bildgebende Verfahren:* Standardröntgen für Arthrosezeichen; MRT zur detaillierten Beurteilung von Knorpelzustand, subchondralen Veränderungen und patellofemoralem Alignment; ergänzende CT bei spezifischen Fragestellungen.
- *Diagnostischer Ansatz:* Kombination aus strukturierter klinischer Untersuchung und gezielter Bildgebung zur differenzierten Diagnosestellung und stadiengerechten und individuell angepassten konservativen oder operativen Therapiekonzepten.

## Abkürzungen

ICRS: International Cartilage Repair Society
LTI: Laterale Trochleainklination
ROM: „Range of motion“
TTPCL: Tuberositas tibiae – „posterior cruciate ligament“
TTTG: Tuberositas tibiae – „trochlear groove“
